# S6Ks isoforms contribute to viability, migration, docetaxel resistance and tumor formation of prostate cancer cells

**DOI:** 10.1186/s12885-016-2629-y

**Published:** 2016-08-05

**Authors:** Camila L. Amaral, Lidia B. Freitas, Rodrigo E. Tamura, Mariana R. Tavares, Isadora C. B. Pavan, Marcio C. Bajgelman, Fernando M. Simabuco

**Affiliations:** 1 Laboratory of Disorders of Metabolism, School of Applied Sciences, University of Campinas, R. Pedro Zaccaria, 1300, sala LA 421, 13484-350 Limeira, São Paulo Brazil; 2 Viral Vector Laboratory, Center for Translational Investigation in Oncology/LIM24, Cancer Institute of São Paulo, School of Medicine, University of São Paulo, São Paulo, Brazil; 3 Brazilian Biosciences National Laboratory, Brazilian National Center for Research in Energy and Materials, Campinas, São Paulo Brazil

**Keywords:** mTOR, S6K, Cancer

## Abstract

**Background:**

The S6 Kinase (S6K) proteins are some of the main downstream effectors of the mammalian Target Of Rapamycin (mTOR) and act as key regulators of protein synthesis and cell growth. S6K is overexpressed in a variety of human tumors and is correlated to poor prognosis in prostate cancer. Due to the current urgency to identify factors involved in prostate cancer progression, we aimed to reveal the cellular functions of three S6K isoforms–p70-S6K1, p85-S6K1 and p54-S6K2–in prostate cancer, as well as their potential as therapeutic targets.

**Methods:**

In this study we performed S6K knockdown and overexpression and investigated its role in prostate cancer cell proliferation, colony formation, viability, migration and resistance to docetaxel treatment. In addition, we measured tumor growth in Nude mice injected with PC3 cells overexpressing S6K isoforms and tested the efficacy of a new available S6K1 inhibitor in vitro.

**Results:**

S6Ks overexpression enhanced PC3-luc cell line viability, migration, resistance to docetaxel and tumor formation in Nude mice. Only S6K2 knockdown rendered prostate cancer cells more sensitive to docetaxel. S6K1 inhibitor PF-4708671 was particularly effective for reducing migration and proliferation of PC3 cell line.

**Conclusions:**

These findings demonstrate that S6Ks play an important role in prostate cancer progression, enhancing cell viability, migration and chemotherapy resistance, and place both S6K1 and S6K2 as a potential targets in advanced prostate cancer. We also provide evidence that S6K1 inhibitor PF-4708671 may be considered as a potential drug for prostate cancer treatment.

**Electronic supplementary material:**

The online version of this article (doi:10.1186/s12885-016-2629-y) contains supplementary material, which is available to authorized users.

## Background

Prostate cancer is the second most frequently diagnosed cancer among men worldwide and the first in developed countries [1]. Although prostate cancer has a good prognosis in its early stages, with nearly all men living at least five years after diagnosis, the 5-year survival rate decreases drastically, to less than 30 %, when it reaches advanced and metastatic stages. This reveals the current urgency to identify factors involved in prostate cancer progression [2].

The S6K proteins are members of the AGC family of serine/threonine kinases and one of the main downstream effectors of the mammalian Target Of Rapamycin (mTOR) protein. In mammals, the S6K family is composed of several proteins encoded by two different genes: RPS6KB1 and RPS6KB2. Due to the alternative use of AUG start codons, each S6K gene generates two distinct isoforms: p70-S6K1, p85-S6K1, p54-S6K2 and p56-S6K2 [3, 4]. More recently, it has been discovered that the splicing factor SF2/ASF acts on S6K1 gene promoting the expression of a novel isoform, p31-S6K1, that lacks most of its catalytic domain [5]. Once activated by mTOR, the S6K proteins are able to phosphorylate targets as rpS6 (ribosomal protein S6), eIF4B (eukaryotic translation Initiation Factor 4B) and eEF2K (eukaryotic Elongation Factor 2 Kinase), promoting protein synthesis and cell growth [3].

Due to their key role in regulating cell growth and proliferation, several studies have shown that S6K genes are amplified in a variety of human tumors, including prostate cancer [6–9]. In fact, S6K is not only overexpressed in prostate cancer, but also is related to its progression [10], making it a potential target for prostate cancer treatment. Despite the high homology shared between S6K1 and S6K2, evidence shows that they might play some distinct cellular functions [11]. Global expression profiles for breast tumors harboring high levels of S6Ks recently revealed that only a few set of genes strongly correlated to both S6K1 and S6K2, suggesting that each protein play different functions in tumorigenesis and cancer progression [12]. However, these differences have been poorly investigated and the major understanding about S6Ks roles in cancer is from studies restricted to p70-S6K1 [13–19].

Here, we aimed to reveal the cellular functions of three S6K isoforms–p70-S6K1, p85-S6K1 and p54-S6K2–in prostate cancer, as well as their potential as therapeutic targets. We show that all isoforms were important for increasing prostate cancer cells proliferation, migration and resistance to docetaxel in vitro. Moreover, S6Ks presented an important effect for tumor progression in vivo. Finally, we demonstrate the potential use of an available S6K1 inhibitor.

## Methods

### Cell culture

Human metastatic prostate cancer cell line PC-3 and the luciferase expressing cell line PC3-luc were cultured in Ham’s F12 (Thermo Scientific) supplemented with 10 % FBS (fetal bovine serum) and 1 % penicillin/streptomycin (Thermo Scientific). Human metastatic prostate cancer cell line DU-145 was cultured in Dulbecco’s Modified Eagle Medium (Thermo Scientific) supplemented with 10 % FBS and 1 % penicillin/streptomycin (Thermo Scientific). Cells were maintained at 37 °C in a humidified atmosphere containing 5 % carbon dioxide.

### Transfection of human cells

Cells were seeded 24 h before transfection. Transfection was performed with Lipofectamine and PLUS reagents (Thermo Scientific). Briefly, DNA and PLUS reagent were diluted in serum free medium and incubated for 15 min at room temperature. Lipofectamine was then diluted in serum free medium, mixed to the DNA solution and incubated for 15 min at room temperature. The cells were washed with serum free medium and the DNA/Lipofectamine complexes were added. After 3 h, the medium was exchanged for medium containing 10 % FBS and the cells incubated for 24 to 72 h.

### Virus production

Lentivirus and retrovirus preparations were generated at Viral Vector Laboratory, Brazilian National Laboratory for Biosciences, Brazilian Center for research in Energy and Materials. Virus were titrated by puromycin selection and counting forming colonies.

### S6Ks RNAi knockdown and overexpression

Lentiviruses expressing shRNA targeting S6K1 and S6K2 human mRNA were produced using pLK0.1 vector (Sigma Aldrich). S6K1 shRNA sequence (TRCN0000022904) has been published before [20]. For S6K2 knockdown, we tested two different shRNA sequences: TRCN0000010539 and TRCN0000199878, identified respectively as shRNA-S6K2-1 and shRNA-S6K2-2. For the control, we used a pLKO.1 plasmid (SHC002) that targets no mammalian genes and was identified as shC.

The retroviral plasmid pBABE-puro was used to perform S6K overexpression. Fragments of S6K isoforms were cloned into *Bam*HI/*Sal*I restriction sites and retroviral-mediated gene transfer was performed as described previously [21, 22].

### Viral transduction

PC3-luc cells were seeded at a density of 8 × 10^3^ cells/well in 96-well plates and incubated for 24 h. Virus particles were added at a low multiplicity of infection (MOI) of 0.3 for lentiviruses and 0.1 for retroviruses in the presence of 8 μg/ml of polybrene. Cell culture medium was changed 24 h after transduction and cells were then selected with 1 μg/mL of puromycin until complete death of control cells.

### Western blotting

Proteins were separated by SDS-PAGE and transferred onto nitrocellulose membranes. Nitrocellulose membranes were blocked in a solution of TBS containing 5 % nonfat dry milk and 0,1 % Tween-20 for 2 h with constant agitation. After blocking, the membranes were incubated with anti-p70-S6K1 (Cell Signaling), anti-S6K2 (Bethyl) or anti-α-tubulin (Calbiochem) antibodies overnight at 4 °C. Membranes were washed with TBS-T (3 times for 15 min) and incubated with horseradish peroxidase-conjugated secondary antibodies (Millipore) for 1 h at room temperature with constant agitation. Bands were visualized using the ECL kit (GE Healthcare). Band densitometry was measured using ImageJ software.

### MTT viability assay

PC3-luc cells with stable S6K knockdown or overexpression were seeded at a density of 10^4^ cells/well in 96-well plates and incubated for 24, 48 and 72 h. Following each period of incubation, 12 mM of 3-(4, 5-methylthiazol-2-yl)-2, 5-diphenyl-tetrazolium bromide (MTT) was added to each well for 4 h. The culture medium was aspirated and the formazan crystals were solubilized with a solution of HCl 1 N:isopropanol (1:25) for 15 min. The optical density of the plates was measured at 570 nm.

### Migration assay

PC3-luc cells with stable S6K knockdown or overexpression were seeded at a density of 5 × 10^5^ cells/well in 24-well plates and incubated until confluence. After that, cell monolayers were scratched in the middle of the wells with a p200 pipette tip and the culture medium was replaced by serum free media [23]. Scratch area was analyzed under light microscope and images were captured right after the scratch (0 h) and after incubation for 24 and 48 h. The scratch area was quantified using ImageJ software.

### Colony-forming assay

Cells were plated at low density (5 × 10^2^ cells / plate) in 60 mm plates and transfected with plasmids pFLAG-p70-S6K1, - p85-S6K1, - p54-S6K2 or pFLAG (empty vector). The cells were incubated at 37 ° C for 10 days. For staining, the cells were washed with PBS and stained with 1 mL of methylene blue dye (3 %) for 30 min. The plates were washed and colonies were counted, excluding colonies smaller than 1 mm in diameter.

### Proliferation assay

Cells were plated in 24-well plates, transfected with plasmids pFLAG- p70-S6K1, - p85-S6K1, - p54-S6K2 or pFLAG (empty vector) and then incubated for up to 6 days with 10 % FBS at 37 ° C. Counts were performed using automated cell counter on days 2, 4 and 6 after transfection.

### Docetaxel resistance assay

Docetaxel resistance assay was performed as previously described by Uzoh et al. [24], with slight modifications. PC3-luc cells with stable S6K knockdown or overexpression were seeded at a density of 8 × 10^5^ cells/well in 24-well plates and incubated for 24 h. The culture medium was then replaced by serum free media and the plates were incubated for 24 h. Afterwards, cells were treated with 30 nM of docetaxel (Sigma Aldrich) for 48 h, which was firstly dissolved in DMSO and then diluted in serum free media. Control cells received serum free media and the vehicle. The number of living cells was counted using an automated cell counter (Thermo Scientific) after Trypan Blue staining.

### In vivo tumor formation assay

All animal experiments were performed in accordance to the internal committee of ethics in animal research of the Faculty of Medicine of the University of São Paulo. One million PC3-luc cells with stable S6K overexpression or knockdown were injected subcutaneously into 7 male athymic Nude mice per group. Tumor growth was measured about three times per week using a caliper rule and calculated according to the formula: ½ × (larger diameter) × (smaller diameter) ^2^ until they reached the maximum volume of 1000 mm^3^.

### Treatment of cells with PF4708671

PC3 and DU145 cells were treated with 10 μM of S6K1 inhibitor PF4708671 [25], by changing medium containing the drug every day, until 6 days. The drug was first dissolved in DMSO and then diluted in medium with serum.

### Statistical analysis

Values presented are means ± standard deviation (SD). Statistical analyzes were assessed by one-way and two-way analysis of variance (ANOVA) followed by Bonferroni’s post-test and performed using GraphPadPrism 5 software. *p*-values < 0.05 were considered significant (*p < 0.05; **p < 0.01; ***p < 0.001).

## Results

### S6K knockdown inhibits cell viability

**Fig. 1:**
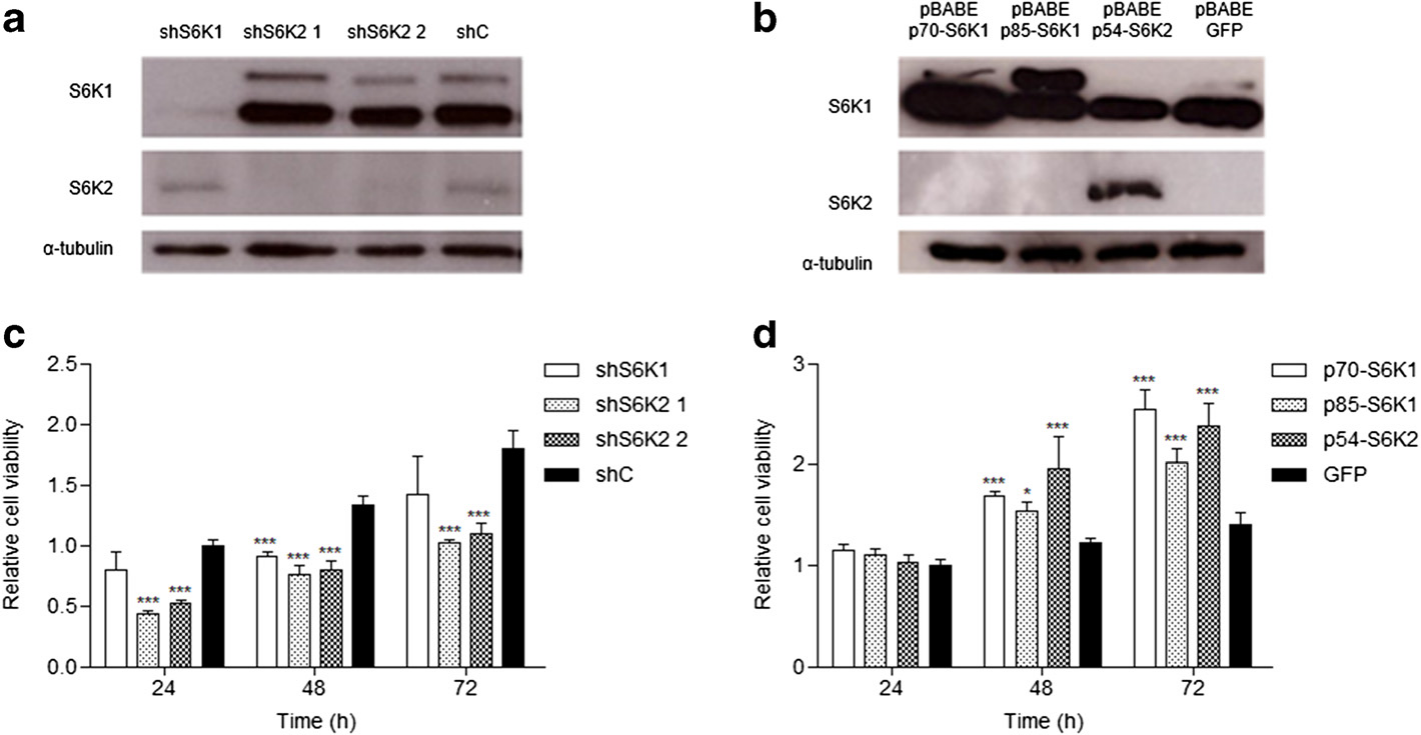
S6Ks increase cell viability in PC3-luc cells. Western blotting analyses of S6K isoforms expression modulation in human prostate cancer cell line PC3-luc transduced with **a** lentiviruses pLKO.1-shRNAs against S6K1 and S6K2 and **b** with retroviruses carrying pBABE construction. S6K1 antibody detects both p85-S6K1 and p70-S6K1 proteins. **c** Relative cell viability in PC3-luc cells with knockdown of S6Ks isoforms and **d** in PC3-luc cells overexpressing S6Ks isoforms. Cells were seeded in 96-well plates at a density of 10^4^ cells/well. After 24, 48 and 72 h, cells were treated with MTT (12 mM) for 4 h and absorbance was measured at 570 nm. *p < 0.05, **p < 0.01, ***p < 0,001, *n* = 3

S6Ks stable knockdown and overexpression in PC3-luc cells were confirmed by western blotting (Fig. 1 a-b). Although some reports indicate that the knockout of one S6K isoform in vivo may cause a compensatory upregulation of the other [26, 27], we observed a slight compensatory effect by S6K1 in the S6K2-1 knockdown in vitro. To investigate the effects of S6Ks knockdown or overexpression in PC3-luc prostate cancer cell line viability, we performed the MTT assay. S6K2 knockdown significantly reduced cell viability in all time periods (Fig. 1c). Similar inhibition was observed in both S6K2 shRNA sequences tested. S6K1 knockdown showed significant reduction only on the second day and appeared to be less relevant than S6K2. However, the overexpression of all three S6K isoforms significantly raised cell viability (Fig. 1d).

### S6K knockdown inhibits cell migration

**Fig. 2:**
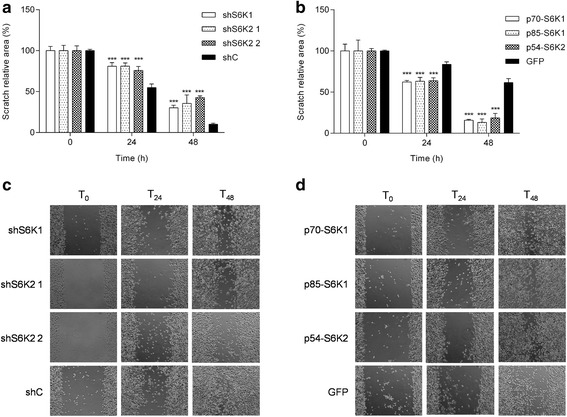
S6Ks increase migration of PC3-luc cells. Cells were seeded in 24-well plates at a density of 5x104 cells/well and incubated until confluence. A scratch was made in the cells monolayer with a pipette tip and cells were washed and incubated in serum free media. The scratch area was measured at 0 (time of scratch), 24 and 48 h. **a** Migration assay in PC3-luc cells with knockdown of S6Ks isoforms. **a**, **b** Scratch relative area of PC3-luc cells overexpressing S6Ks isoforms. **c**, **d** Representative images of scratch area taken at 0, 24 and 48 h of PC3-luc cells with S6K knockdown and overexpression, respectively. *p < 0.05, **p < 0.01, ***p < 0,001, *n* = 3

Next we investigated if S6K isoforms also impacted the migration capacity of prostate cancer cells (Fig. 2). Both S6K1 and S6K2 knockdown resulted in a significant decrease in cell migration. Again, no difference was observed between both S6K2 shRNA sequences. In contrast, as expected, the overexpression of S6K isoforms significantly increased scratch area closure.

### S6K2 expression is related to docetaxel sensitivity

**Fig. 3:**
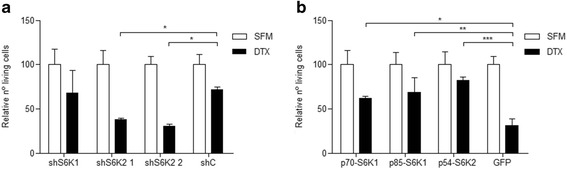
p54-S6K2 knockdown increases cell death in response to docetaxel in PC3-luc cells. Cells were seeded in 24-well plates at a density of 8x104 cells/well. After 24 h, cells were subjected to serum free media conditions for 24 h and then treated for 48 h with docetaxel (30 nM). Living cells were counted and expressed as percentages. Results presented are means of two independent experiments. Each experiment was performed in triplicates. SMF: serum free media; DTX: docetaxel. *p < 0.05, **p < 0.01, ***p < 0,001, *n* = 2

We investigated whether S6Ks might play a role in prostate cancer chemotherapy resistance. We treated PC3-luc with docetaxel, a chemotherapeutic drug widely used in hormone-refractory prostate cancer treatment [28]. As expected, docetaxel treatment reduced the number of living cells in all groups when compared to untreated cells (Fig. 3). However, the reduction was significantly greater in cells with S6K2 knockdown (Fig. 3a). When overexpressed, all S6Ks isoforms showed significantly less reduction in the number of living cells than the GFP control (Fig. 3b).

### S6K overexpression enhances tumor growth in Nude mice

**Fig. 4:**
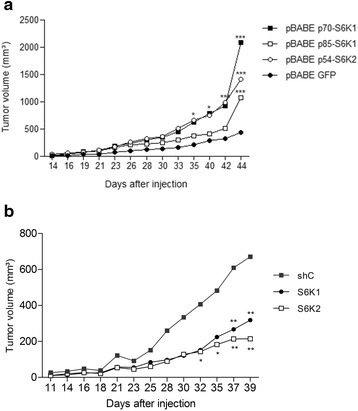
S6K isoforms increase tumor growth in Nude mice. **a** Tumor growth curves of Nude mice injected subcutaneously with PC3-luc cells overexpressing S6K isoforms (p70-S6K1, p85-S6K1 and p54-S6K2) or GFP control. Tumors measurements began 14 days after injection and proceeded until day 44. **b** Tumor growth curves of Nude mice injected subcutaneously with PC3-luc cells with knockdown for S6K1 and S6K2 isoforms or shRNA control. Tumors measurements began 11 days after injection and proceeded until day 39. * *p* < 0,05, ** *p* < 0,01 e *** *p* < 0,001; *n* = 7

After having determined that S6Ks are involved in some prostate cancer features in vitro, we investigated their tumorigenic ability in vivo, by injecting PC3-luc cells overexpressing S6K isoforms or knocked down for S6K1 or S6K2 subcutaneously into the flanks of male athymic Nude mice and measuring tumor growth. Tumors started growing about 10 days after injection and from this moment tumor volume was assessed for about 30 days. Animals injected with PC3-luc cells overexpressing S6K isoforms, particularly p70-S6K1, showed significantly greater tumor growth than those injected with cells harboring GFP control (Fig. 4a). Conversely, animals injected with PC3-luc cells containing the knockdown of S6K1 or S6K2 presented significantly reduced tumor growth in vivo (Fig. 4b).

### S6K1 inhibitor PF47086701 decreases proliferation and migration of PC3 prostate cancer cell line

**Fig. 5:**
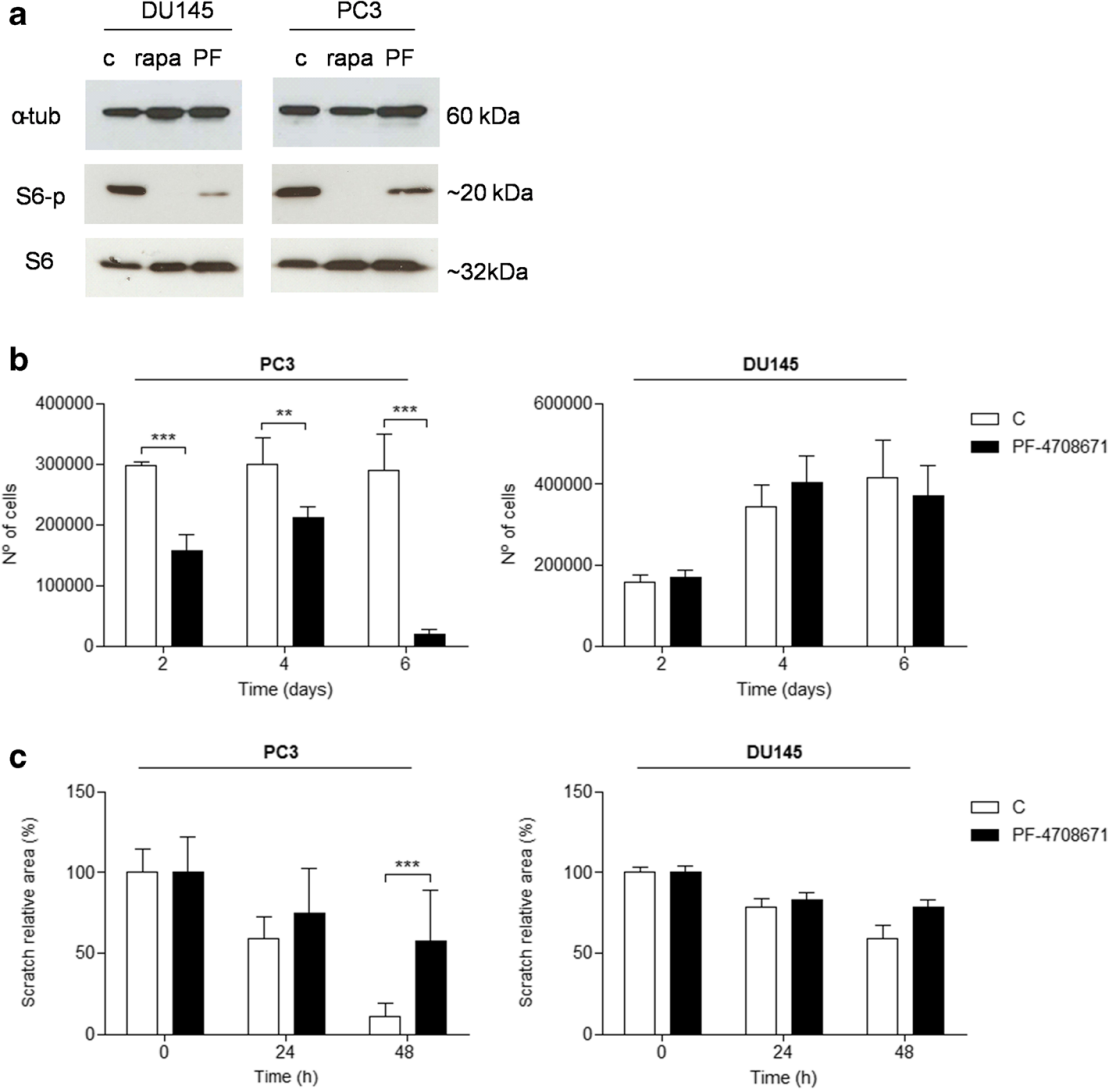
S6K1 inhibitor PF47086701 inhibits cell proliferation and migration of prostate cancer cells. **a** Western blotting analysis of PF47086701 efficiency. **b** Proliferation assay in DU145 and PC3 cell lines treated with PF47086701. **c** Scratch relative area of DU145 and PC3 cells treated with PF47086701. *p < 0.05, **p < 0.01, ***p < 0,001, *n* = 3

To check the efficiency of S6K1 inhibitor in prostate cancer cells DU145 and PC3, western blotting was performed in order to evaluate the S6 phosphorylation status (Fig. 5a). Rapamycin was used in this experiment as control. PF47086701 was able to reduce S6 phosphorylation in both DU145 and PC3 cells lines, although its effect was more discrete when compared to rapamycin, which completely abolished S6 phosphorylation (Fig. 5a). In a proliferation assay, DU145 and PC3 cells were plated and counted after treatment with PF47086701 (10 μM). Only PC3 cells showed a decrease in the number of cells when treated with the inhibitor of S6K1 (Fig. 5b). In a migration assay, the scratched monolayers were treated with serum-free medium and PF4708671 (10 μM) daily. The areas were measured within 48 h after the scratch. PC3 cells showed a significant decrease in migration compared to control in the presence of S6K1 inhibitor (Fig. 5c).

To compare the effects of S6Ks overexpression in DU145 and PC3 cell lines, we transiently transfected them with pcDNA-FLAG vector carrying p70-S6K1, p85-S6K2 and p54-S6K2 genes (Additional file 1: Figure S1A). The results show that in both cell lines p85-S6K1 isoform enhanced the cellular proliferation (Additional file 1: Figure S1B). Colony formation assay also indicated that cells with overexpression of p85-S6K1 showed increased number of colonies (Additional file 1: Figure S1C). These results point an important role of S6K1 isoform in prostate cancer.

## Discussion

S6K proteins are well known effectors of mTOR and major regulators of protein synthesis and cell growth [4]. They are found overexpressed in several human tumors and are correlated with poor prognosis [10, 29, 30]. It has already been reported that silencing mTOR decreases prostate cancer cell proliferation and colony formation [31]. Here we show that S6Ks overexpression enhances proliferation and clonogenic ability of human metastatic prostate cancer cell lines. Next, we evaluated the effects of S6K knockdown and overexpression on prostate cancer cells viability, migration and resistance to decetaxel treatment.

Recently, Du et al. (2014) showed that mTOR knockdown by lentivirus mediated shRNA significantly reduces cell viability in prostate cancer metastatic cell lines LNCaP and C4-2B. As expected, we not only observed that S6K knockdown produced similar effects on PC3-luc cells, but also reported an expressive increase in cell viability determined by S6K overexpression. Interestingly, we found that although the effects of p70-S6K1 and p54-S6K2 overexpression were quite similar, S6K1 knockdown had no impact on cell viability. A possible explanation is that S6K1 knockdown is compensated by S6K2 isoform, masking the effects on cell viability.

We also reported that S6K expression is positively related to an increase in PC3-luc cells migration. Since tumor cell motility is a fundamental part of the metastatic cascade, our data imply an involvement of all S6K isoforms in raising metastatic capability of PC3-luc cells. In fact, p70-S6K1 has been described as a regulator of cell motility [32] and several human tumorigenic cell lines, such rhabdomyosarcoma (Rh1 and Rh30), breast cancer (MDA-MB-468) and cervical adenocarcinoma (HeLa) were shown to alter their motility and invasion capabilities in response to rapamycin and these phenomena were regulated by mTOR through S6K1 and 4E-BP1 [33]. p70-S6K1 can directly bind to F-actin and is localized at the actin arc of migrating cells [34, 35]. In ovarian cancer cells, p70-S6K1 regulates cytoskeleton organization and cell migration by activating the GTPases Rac1 and Cdc42, involved, respectively, in dictating forward movement and migration’s direction [34]. Indeed, p70-S6K1 knockdown ovarian cancer cells migrate less and exhibit reduced directional persistence [34]. Our results show that S6K1 promotes cell migration in PC3-luc cell line and that S6K2 is also involved in the regulation of this process.

S6K2 knockdown was also effective in raising docetaxel sensitivity in PC3-luc cells. It has been reported that S6K2 is activated by FGF-2 (Fibroblast Growth Factor-2) and phosphorylates PDCD4, a repressor of the translation of anti-apoptotic proteins such as XIAP and Bcl-xL [36]. PDCD4 phosphorylation by S6K2 causes its degradation and leads to survival and chemoresistance in lung cancer cells [36, 37]. Although studies relating S6Ks to chemotherapy resistance are scarce, several reports indicate that mTOR is involved in drug resistance against chemotherapy [38–43]. Niu et al. [44] showed that mTOR inhibition by rapamycin raises apoptosis and sensitivity to docetaxel induced cytotoxity in several human metastatic lung cancer cell lines treated with different concentrations of docetaxel. Treatment with NVP-BEZ235-a PI3K/mTOR dual inhibitor–was able to sensitize human castration resistant prostate cancer cell lines C4-2 and C4-2AT6 resistant to docetaxel, indicating that mTOR inhibition can even overcome chemoresistance in castration resistant prostate cancer [45]. However, the specific role of mTOR in developing chemotherapy resistance is still poorly understood, yet of current clinical importance. Here, we show that docetaxel resistance in PC3-luc cell line may be at least in part mediated by S6K, especially p54-S6K2.

The oncogenic role of S6K was also confirmed in vivo. The overexpression of all S6K isoforms was able to enhance tumor formation in Nude mice and the knockdown of S6Ks isoforms was able to reduce tumor growth in vivo. This result corroborates to Du et al. [31], that demonstrated that Nude mice injected with human metastatic prostate cancer cell line C4-2B with mTOR knockdown presented a greater reduction in tumor volume when compared to control group. As it was shown that p70-S6K1 played a major role in increasing tumor formation in vivo, we decided to test the effectiveness of a novel S6K1 inhibitor, PF-4708671.

We demonstrated that targeting S6K1 with PF-4708671 provided reduced cell migration and proliferation in PC3 cells, but had no effect on DU145 cell line (Fig. 5). A possible explanation for this difference is that PC3 cells, but not DU145, are PTEN null. PTEN deletion leads to increased PI3K activity and therefore hyperactivation of mTORC1/S6K [46]. Thus, it is possible that the mechanisms inducing proliferation and migration in PC3 cells are more related to S6K activity than in DU145 cells and, therefore, more susceptible to PF-4708671 effect.

PF-4708671 is a cell-permeable piperazinyl-pyrimidine compound that specifically inhibits p70-S6K1 with a Ki of 20 nM and IC_50_ of 160 nM and exhibits no significant inhibition of S6K2 or other AGC kinases [25], which may explain the reduced effect to abolish S6 phosphorylation comparing to rapamycin (Fig 5a), since S6K2 may still act on S6 protein. Due to its recent development, there are only a few reports regarding PF-4708671 use in anti-cancer therapies [3, 25]. S6K1 inhibition by PF-4708671 was shown to sensitize resistant colorectal cancer cells to selumetinib [47], to decrease cell migration and invasion of MDA-MB-231 human breast cancer cell line [48] and to inhibit cell invasion and proliferation in human lung cancer cell lines and tumorigenesis in Nude mice [49]. Hence, our results ratify that PF-4708671 might be a novel potential adjuvant in metastatic prostate cancer drug treatment.

## Conclusions

In summary, our study demonstrated that S6K overexpression enhances cell viability, migration and resistance to docetaxel in PC3-luc prostate cancer cell line and tumor volume in Nude mice. In addition, we showed that the often neglected S6K2 is also involved in these processes and might be a potential target to restore docetaxel sensitivity in advanced prostate cancer. The S6K1 inhibitor PF-4708671 was particularly effective in reducing cell migration of PC3 and DU145 cell lines, suggesting that it could represent a possible adjuvant to prevent prostate cancer progression to its advanced state.

## Abbreviations

eEF2K, eukaryotic Elongation Factor 2 Kinase; eIF4B, eukaryotic translation Initiation Factor 4B; FBS, fetal bovine serum; mTOR, mammalian Target Of Rapamycin; MTT, 12 mM of 3-(4, 5-methylthiazol-2-yl)-2, 5-diphenyl-tetrazolium bromide; rpS6, ribosomal protein S6; S6K, S6 Kinase.

## Additional file

Additional file 1: Figure S1. Transient overexpression of different S6Ks isoforms in DU145 and PC3 cell lines. (A) Western blotting analysis of transfection efficiency. (B) Proliferation assay in DU145 and PC3 cell lines transfected with p70-S6K1, p85-S6K1 and p54-S6K2. (C) Colony formation assay of DU145 and PC3 cells transfected with p70-S6K1, p85-S6K1 and p54-S6K2. **p* < 0.05, ***p* < 0.01, ****p* < 0,001, *n* = 3. (TIF 2571 kb)
