# Supplementary figures and images for: S6Ks isoforms contribute to viability, migration, docetaxel resistance and tumor formation of prostate cancer cells
Source: BMC Cancer. 2016 Aug 5;16:602. doi: 10.1186/s12885-016-2629-y (PMC4974797; doi:10.1186/s12885-016-2629-y)

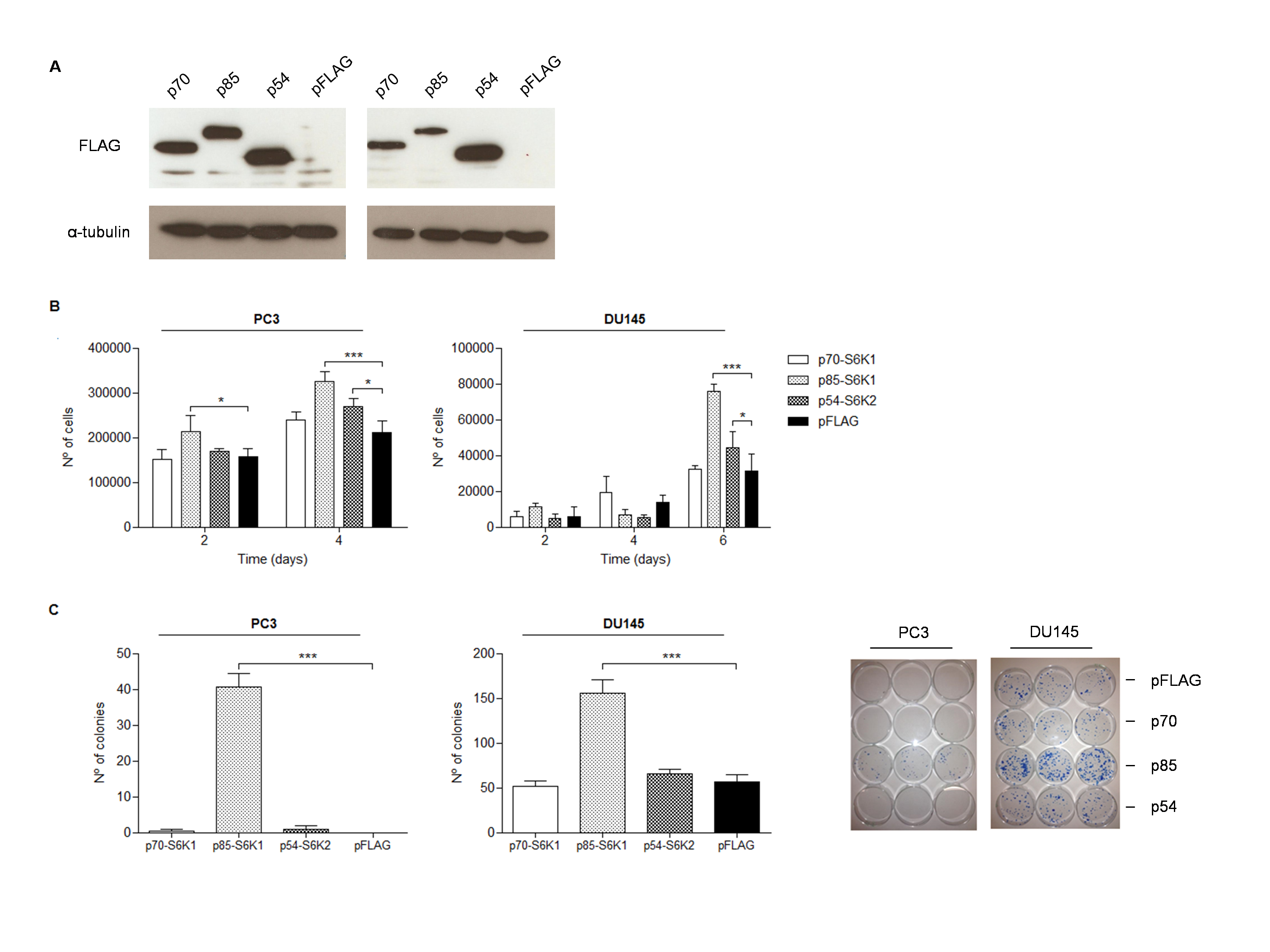

Supplement: Additional file 1: Figure S1. — Transient overexpression of different S6Ks isoforms in DU145 and PC3 cell lines. (A) Western blotting analysis of transfection efficiency. (B) Proliferation assay in DU145 and PC3 cell lines transfected with p70-S6K1, p85-S6K1 and p54-S6K2. (C) Colony formation assay of DU145 and PC3 cells transfected with p70-S6K1, p85-S6K1 and p54-S6K2. *p < 0.05, **p < 0.01, ***p < 0,001, n = 3. (TIF 2571 kb) [file 12885_2016_2629_MOESM1_ESM.tif]
